# Wegener Granulomatosis Revealed by Pleural Effusion

**DOI:** 10.1155/2009/164395

**Published:** 2010-02-04

**Authors:** Anne-Claire Toffart, François Arbib, Sylvie Lantuejoul, Jean-François Roux, Vincent Bland, Gilbert Ferretti, Samia Diab

**Affiliations:** ^1^Clinique de Pneumologie, Pôle Médecine Aiguë Communautaire, Centre Hospitalier Universitaire Albert Michallon, 38043 Grenoble cedex 09, France; ^2^Département de Pathologie, Centre Hospitalier Universitaire Albert Michallon, 38000 Grenoble, France; ^3^INSERM U 823, Université Joseph Fourier, 38000 Grenoble, France; ^4^Chirurgie Générale et Thoracique, Clinique des Cèdres, 38130 Echirolles, France; ^5^Cabinet Médical Anatomie Cytologie Pathologiques Ciapa Bland Muller, 38000 Grenoble, France; ^6^Clinique Universitaire de Radiologie et Imagerie Médicale, Centre Hospitalier Universitaire Albert Michallon, 38043 Grenoble cedex 09, France

## Abstract

Pulmonary signs are common in Wegener's granulomatosis (WG). However, an initial presentation including pleural effusion has not been described. We describe a case of WG in which pleural effusion was the first clinical manifestation. A 45-year-old man with dorsal pain presented with pleural thickening and effusion, and a visible nodule on a thoracic scan. A dense chronic inflammatory infiltrate was obtained by pleural biopsy and an open lung biopsy revealed necrotizing granulomatous vasculitis. Serologies were positive for antineutrophil cytoplasmic antibodies and antiproteinase 3 antibodies. A diagnosis of WG was conducted and the patient was started on cyclophosphamide and methylprednisolone as an initial treatment, with a favorable evolution. Although pleural effusion is rarely described in WG, this pathology must be considered in the presence of this clinical manifestation.

## 1. Introduction

Wegener's granulomatosis (WG) is an anti-neutrophil cytoplasmic antibody- (ANCA-) associated granulomatous vasculitis of small and medium-sized vessels. The clinical signs vary but pulmonary manifestations are common. Thoracic lesions generally consist of intrapulmonary nodules, which are mostly cortical and excavated, with irregular margins. Parenchymal consolidation and pleural involvement are less frequent. However, to our knowledge, an initial presentation including pleural effusion has rarely been described.

## 2. Case Report

**Figure 1:**
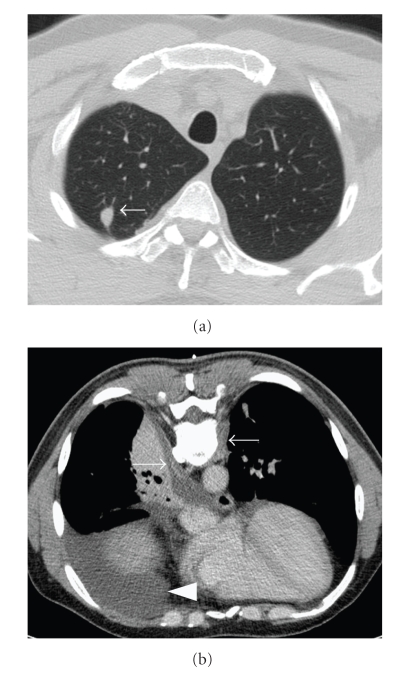
(a) CT scan revealing a nonspecific nodule in the right upper lobe (arrow). (b) Contrast enhanced CT in prone position shows right pleural effusion (arrowhead) and bilateral sub pleuralthickening (arrow) in the region of the 9th vertebrae.

**Figure 2:**
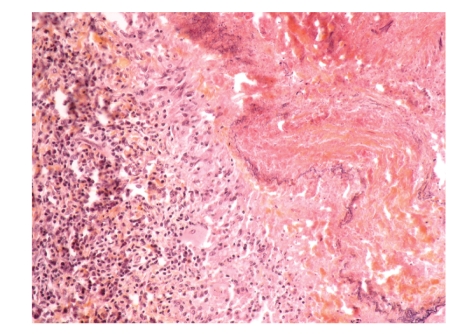
At histological examination, the pulmonary nodule was centered by an area of necrosis, surrounded by a palissade of histiocytes, sometimes giant and multinucleated. Elastic stain enlightens the elastic lamnia, partially destroyed by this granulomatous inflammation (Elastic Stain, original magnification ×200).

A 45-year-old man with an unremarkable medical history presented in April 2008 with severe dorsal pain requiring morphine. He did not drink or smoke. His occupational history revealed a 25-year employment as a metalworker. Physical examination was normal, except for asthenia and neurological signs (right hemithoracic pain, bilateral paresthesia, and dysesthesia C8-D1). An initial chest X-ray was not informative. A contrast enhanced CT revealed a nonspecific 1.5 cm pulmonary nodule in the right upper lobe (Figure 1(a)), right pleural effusion, and bilateral latero-spinal subpleural thickening in the region of the 9th vertebra (Figure 1(b)). The white blood cell (WBC) count was 12000/mm^3^, eosinophils were normal, renal function was normal, and minimal cholestasis was found. Inflammation of the right lower lobe bronchus was observed on bronchoscopy, but biopsies were not informative. A thoracocenthesis was performed and revealed a sterile exudative lymphocytic pleural effusion (lymphocytes 85%, neutrophils 10%, and monocytes 5%), without malignant cells. It was decided to obtain pleural biopsies during surgical resection of the nodule. Histological examination of the pleura revealed a dense inflammatory infiltrate predominating at the periphery of the vessels, composed mainly of lymphocytes and plasma cells. No true vasculitis could be demonstrated within the pleura. The pulmonary specimen contained a central necrotic nodule, surrounded by palissading histiocytes (Figure 2). Some of the histiocytes were multinucleated and formed true granulomas within the wall of the adjacent pulmonary artery. Serologies were strongly positives for ANCA (ANCA+++) and antiproteinase 3 (APR3) antibodies (129 IU; normal <30). No renal biopsy was performed as urine analysis and renal function were normal. Skin examination revealed nonspecific ulceration on the patient's forehead. A sinus scan revealed septum perforation and ethmoidal sinus thickening. 

A diagnosis of WG was made based of the histopathology and ANCA positivity. Initial treatment consisted of intravenous methylprednisolone followed by oral prednisolone and cyclophosphamide. After 4 months, azathioprine was started with decreasing doses of glucocorticoids. The patient improved and the peripheral neurological signs decreased. The thoracic scan normalized (disappearance of pleural effusion and no residual thickening) and antibody levels decreased (ANCA +, APR3 (6 IU)).

## 3. Discussion

The presence of pleural effusion suggested a diagnosis of purulent pleurisy (inflammatory syndrome), tuberculosis (lymphocytes in pleural effusion), or malignant disease due to the asthenia and chronic thoracic pain. Parietal or neurological conditions such as epiduritis or bone injuries were also considered in the differential diagnosis because of the asthenia and pain. Parenchymatous pulmonary manifestations are common in WG (48%–73% at presentation, 85%–92% during the disease), but pleural damage is rare, especially as a first sign. Pleural lesions are present in only 10% of WG patients with initial pulmonary involvement [3]. Pleural thickening and pleural effusion are the most frequent signs, and little is known about their histology. Pleural thickening may result from previous effusion or represent cicatricial changes resulting from nodular inflammatory lesions extending into the pleura [4]. Pleural effusion is often unilateral, characterized by its low volume and important pachypleuritis [1, 2]. Pleural tap and biopsy are difficult. The pleural exudate is associated with hypoglycopleury and predominant neutrophils and is sterile except when the nodule is infected and ruptured within the pleural space [3]. Association with other pulmonary damage (excavated or nonexcavated nodule, infiltrate) on imagery guides the diagnosis. Kumasaka et al. [5] described one case of small vessel vasculitis limited to pleuropulmonary manifestations, possibly induced by endotoxin. Immunohistochemistry revealed the expression of IL-1*β* and VCAM-1, which may have caused activation of monocytes and endothelial cells within the vasculitic lesions [5]. One of the two cases of isolated and inaugural pleurisy described in WG was characterized by an eosinophilic effusion without peripheral blood eosinophilia [2]. These cases are presented in Table 1. In our case, the patient developed pleural thickening, pleural effusion, and an isolated nodule simultaneously. The final diagnosis was based on clinical, radiological, histological, and biological arguments.

It should be pointed out that pleural effusion can be a manifestation of vasculitis and WG, and that this diagnosis should be considered if infectious and malignant diseases have been ruled out. This disease location seems to respond to immunosuppressive drugs as well as other localizations of WG.

## Figures and Tables

**Table 1:** Cases published with similar initial pleural effusion in Wegener's granulomatosis.

	Blundell et al. [1]	McCann et al. [2]	Toffart et al.
Clinical presentation	64-year-old woman	65-year-old man	45-year-old man
	Fever	Chest pain, dyspnoea	Dorsal pain
Initial chest X-ray	Left pleural effusion	Left pleural effusion	Non informative
Blood biology	WBC 13000/mm^3^	WBC <12100/mm^3^	WBC 12000/ mm^3^
		No eosinophilia	No eosinophilia
Fluid analysis	Unsuccesfull	Exsudate: protein 53 g/L	Exsudate: protein 49.7 g/L
		WBC count: 7.69/mm^3^	WBC count: 35/mm^3^
		(i) 25% eosinophils	(i) 85% lymphocytes
		(ii) 37% neutrophils	(ii) 10% neutrophils
		(iii) 34% lymphocytes	(iii) 5% monocytes
